# Indicators of chronic noncommunicable diseases in women of reproductive age that are beneficiaries and non-beneficiaries of Bolsa Família

**DOI:** 10.1590/1980-549720190012.supl.1

**Published:** 2019-10-07

**Authors:** Regina Tomie Ivata Bernal, Mariana Santos Felisbino-Mendes, Quéren Hapuque de Carvalho, Jill Pell, Ruth Dundas, Alastair Leyland, Mauricio Lima Barreto, Deborah Carvalho Malta

**Affiliations:** I Graduate Program, School of Nursing, Universidade Federal de Minas Gerais - Belo Horizonte (MG), Brazil; II Institute of Health and Wellbeing, University of Glasgow - Glasgow, Scotland; III Medical Research Council/Chief Scientific Office (MRC/CSO) Social and Public Health Sciences Unit, University of Glasgow – Glasgow, Scotland; IV Center for the Integration of Health Knowledge and Data, Gonçalo Moniz Institute, Oswaldo Cruz Foundation - Salvador (BA), Brazil

**Keywords:** Socioeconomic factors, Noncommunicable diseases, Women’s Health, Health surveys, Hematologic tests

## Abstract

**Objective:**

To evaluate the prevalence of noncommunicable disease (NCD) indicators, including laboratory tests, in the population of Brazilian women of reproductive age, according to whether or not they receive the *Bolsa Família* (BF) benefit.

**Methods:**

A total of 3,131 women aged 18 to 49 years old who participated in the National Health Survey (*Pesquisa Nacional de Saúde*) laboratory examination sub-sample were considered. We compared indicators among women of reproductive age (18 to 49 years old) who reported receiving BF or not, and calculated prevalence and confidence intervals, using Pearson’s χ^2^.

**Results:**

Women of reproductive age who were beneficiaries of BF had worse health outcomes, such as a greater occurrence of being overweight (33.5%) and obese (26.9%) (p < 0.001), having hypertension (13.4% versus 4.4%, p < 0.001), used more tobacco (11.2% versus 8.2%, p = 0.029), and perceived their health as worse (6.2% versus 2.4%, p < 0.001).

**Conclusion:**

Several NCD indicators were worse among women of childbearing age who were beneficiaries of BF. It should be emphasized that this is not a causal relationship, with BF being a marker of inequalities among women. The benefit has been directed to the population with greater health needs, and seeks to reduce inequities.

## Introduction

Chronic noncommunicable chronic diseases (NCDs) are responsible for a high number of premature deaths, the loss of quality of life, and a high degree of limitation for individuals. Furthermore, they cause negative economic impacts on families, communities and society in general, which results in worsening social inequities and poverty^1^.

The NCD epidemic has most affected low-income people because they are more exposed to risk factors and have less access to health services^2^. There are important differences in the distribution of morbidity and mortality of these diseases according to socioeconomic factors such as education, occupation, income, gender and ethnicity, causing differential access to services and consumption patterns, among other things^2^.

In Brazil, NCDs are also a major health problem, accounting for 75% of the causes of death and, although they affect individuals from all socioeconomic strata, those from vulnerable groups, such as the elderly and those with low levels of education and income, are hit the hardest^3^. Additionally, studies demonstrate a relationship between social determinants, poor socioeconomic conditions and poor health outcomes with greater susceptibility to develop NCDs and their comorbidities, in addition to higher mortality rates^2,4,5^.

Data from the National Household Sample Survey (*Dados da Pesquisa Nacional por Amostra de Domicílio* - PNAD) in 2003 already showed a high prevalence of NCDs in the female population and in other individuals with low levels of education^6^. The National Health Survey (*Pesquisa Nacional de Saúde* - PNS) also indicates that, among the NCDs analyzed in the survey, most were reported by women. Additionally, people with chronic diseases reported worse self-evaluated health^7^. This may be due to the fact that women use health services more often^8^ and also because they are more attentive to their health^8^.

On the other hand, few studies address the magnitude of NCDs among women of reproductive age ^9–11^, since the predominant approach to research on this specific group is related to reproductive issues such as family planning, prenatal care, prevention and screening for gynecological cancers. Research dealing with NCDs shows how much these diseases increasingly affect women^10–12^, even though they are young. Consequently, they also affect reproductive issues^13^.

There is still a significant gap in research regarding possible inequities, i.e. whether women with unfavorable socioeconomic conditions are more susceptible to NCDs and their risk factors. A previous study with the Surveillance of Risk Factors and Protection for Chronic Diseases by Telephone Survey (*Vigilância de Fatores de Risco e Proteção para Doenças Crônicas por Inquérito Telefônico* - Vigitel) found that women of reproductive age with low levels of education were more inactive, and had higher levels of smoking and hypertension^11^. Cardiovascular diseases are also treated and prevented to a lesser extent among women, especially those who are in positions of social vulnerability^14^. Moreover, due to gender inequality, sexist practices place women in unfavorable situations, which are further aggravated by economic inequality^15,16^.

The Bolsa Familia (BF) program, a conditional cash transfer program (*um programa de transferência condicionada de renda* - PTCR), was created in Brazil in 2003 with the objective of increasing guaranteed social protection and reducing poverty. It is an act of positive discrimination that aims to break the intergenerational cycle of poverty, and reduce vulnerabilities and social inequalities^17,18^. Most of the beneficiaries of BF are poor and socially disadvantaged women^19,20^. The program aims to reduce social inequities and focus on social determinants and the context in which everyone lives^4,22^.

We hypothesized that women receiving BF would have less access to health services and worse health indicators. Thus, this study aimed to evaluate the prevalence of NCD indicators, including laboratory tests, in the population of Brazilian women of reproductive age according to whether they receive BF. It is believed that this unprecedented assessment can identify inequalities among women and provide more knowledge of the occurrence of these diseases in this specific subset of the population.

## Methods

This research is a descriptive cross-sectional study based on secondary PNS data. The PNS is a household survey that is part of the Integrated Household Survey System of the Brazilian Institute of Geography and Statistics (*Instituto Brasileiro de Geografia e Estatística* - IBGE)^23,24^.

The PNS uses the three-stage cluster sampling process. Census sectors or sets of sectors form the primary sampling units (PSUs); households form the second stage units; and residents 18 and older form the third stage units. 60,202 individuals with a response rate of 86% were interviewed and, because it is a complex sample, expansion factors or sample weights were defined for the PSUs, for the households, for all of their residents, as well as for the selected resident. More details on sampling and data collection are available in other publications^23^.

A laboratory research module was included in the PNS and a subsample containing 25% of the census tracts was defined. However, the laboratory subsample obtained comprised 8,952 people. Several factors caused sample losses, such as the hired laboratory having difficulty finding addresses, the refusal of the selected resident to perform biological material collection, and the long period of time that elapsed between the interview and the laboratory collection. Post-stratification weights according to gender, age, education and region were used to correct possible biases^23,24^ in the statistical analyses.

The concept of women of childbearing age or reproductive age refers to those aged 15 to 49 years old^25,26^, however, the present study only analyzed data from women aged 18 to 49 years old, since the cutoff point used in the PNS is an adult population aged 18 years or older. The sociodemographic distributions of the sample were detailed.

Regarding laboratory analysis, the PNS subsample was 8,952 respondents. Therefore, the current study included 3,131 women aged 18 to 49 years old who participated in this laboratory sub-sample.

Data referring to the individual questionnaire, as well as laboratory data, were used to compose the CNCD indicators for this study.

HbA1c was collected in a tube with ethylenediamine tetraacetic acid (EDTA) and dosed by high pressure liquid chromatography (HPLC) by ionic exchange. The World Health Organization (WHO) cut-off point was used, and the American Diabetes Association recommends HbA1c ≥ 6.5% for the diagnosis of diabetes mellitus ^27,28^.

Total cholesterol, low density lipoprotein (LDL) and high density lipoprotein (HDL) were collected in a gel tube. The following cutoff points for total cholesterol (TC) and fractions were established: TC ≥ 200 mg/dL; LDL ≥ 130 mg/dL and HDL <40 mg/dL, following the clinical treatment parameters recommended by the Adult Treatment Panel III^29^.

Serum creatinine was collected in a gel tube and dosed by the Jaffé method without deproteinization. For the dichotomous analysis, the values ≥ 1.3 mg/dL were considered to be altered. The glomerular filtration rate (GFR) was calculated for creatinine by using predictive equations that utilize correction factors (age, gender, race and weight)^30,31^. Estimated GFR <60 mL/min/1.73 m^2^ was calculated based on separate creatinine for women.

Red series tests were analyzed, and at this time anemia was considered when hemoglobin was <12 g/dL, the WHO standard^32^.

Urine samples were collected at different times throughout the day. Urinary sodium was measured using the sensitive electrode method. The frequency of the population above the 75th percentile of salt intake was taken into account.

For laboratory tests, prevalence, 95% confidence interval (95% CI), and age-adjusted prevalence ratio (PR) were calculated, comparing whether or not they received BF.

Receipt of BF was taken from question F012 of the PNS questionnaire: “In July (reference month), does any resident of this household receive income from the Bolsa Familia Program?”. In the PNS laboratory database, IBGE incorporated variables related to NCDs, which were analyzed here, and compared the prevalence and 95% CI among women of reproductive age (18 to 49 years) who said they received BF or not. The indicators included in this study were risk and protection factors against NCDs:

- anthropometric measurements: weight and height were measured by scale and anthropometers, and body mass index (BMI) was calculated - overweight: BMI between ≥ 25 and <30 kg/m^2^; obese: BMI ≥ 30 kg/m^2^);
- smokers: report smoking regardless of the number of cigarettes;
- consumption of excess meat fat: eats red meat with visible fat or chicken with skin;
- regular consumption of soft drinks or artificial juices five or more days a week;
- bean consumption five or more days a week;
- alcohol abuse, five or more doses on one occasion in the last 30 days;
- self-evaluated health: three categories were classified to assess health status - very good, fair and poor.

The following morbidity indicators previously diagnosed by the physician were considered:

- arterial hypertension;
- diabetes;
- cholesterol;
- arthritis or rheumatism;
- renal insufficiency.

The indicators of access to health services included:

- mentioning whether or not they had health or dental insurance;
- saying they have looked for health services in the last year;
- hospitalization in the last 12 months.

Thus, the study estimated and compared prevalences and 95% confidence intervals (CI) using Pearson’s χ^2^, and analyses were performed using Stata, version 13. The PNS questionnaire and the variables have already been published in previous publications, and more details can be found in other publications^8,23^.

As provided in the research protocol and in the Ethics Committee, all test results were reported to the user by the laboratory, and in case of indicative results, the participants were advised to seek medical attention in the public health system. In cases of extreme risk, participants were contacted directly by the laboratory or the Ministry of Health, encouraging participants to seek out immediate care.

The PNS was approved by the National Research Ethics Commission, under No. 328,159, of June 26, 2013. All individuals were consulted, informed and agreed to participate in the research.

## Results

Among the 3,131 women studied, 924 (23.3%) were beneficiaries of the BF program. Regarding sociodemographic characteristics, 1,087 (40.8%) were between 18 and 29 years old, 1,769 (50.9%) were between 30 and 44 years old, and 275 (8.3%) were between 45 and 49 years old. Women receiving BF were less educated, 49.6% had from zero to eight years of schooling, while among non-beneficiaries only 16.2% had up to eight years of schooling and more than half had 12 or more years of schooling. Most women receiving BF self-reported as light-skinned black and dark-skinned black, and the majority of non-beneficiaries said they were white (51.1%). Women receiving BF were more concentrated in the Northeast (50.2%), followed by the Southeast (26.7%). Most non-beneficiaries lived in the Southeast (45.6%) (Table 1).

Table 2 shows the prevalence of indicators for NCDs, and there was a higher occurrence of diseases among women who benefit from BF. The beneficiaries had a higher prevalence of being overweight (33.5%) and being obese (26.9%) (p <0.001). They also showed higher tobacco consumption, but lower alcohol consumption. Bean consumption was higher (75%) among BF beneficiaries (p <0.001). Missing information on risk factors ranged from 0.09 to 5%, data not shown.

Women who receive the benefit are about three times as likely as non-beneficiaries to rate their health as poor (p <0.001), and the vast majority of women who benefitted from BF had no health or dental insurance (94.4%) (p <0.001). It is also worth noting that the beneficiary women had a higher prevalence of hypertension, especially during pregnancy (p <0.001). It was found that 15% of respondents did not have information on high cholesterol, and 10% did not have information on diabetes, data not shown (Table 3).

Regarding the laboratory tests, it was observed that although beneficiary women had a higher prevalence of renal failure, increased creatinine, HDL, diabetes, and anemia, the difference was only significant for HDL cholesterol (Table 4). That is, women who receive BF had 41% higher prevalence of HDL cholesterol <40 mg / dL than those who did not receive the benefit.

## Discussion

The results of this study show that women of reproductive age who benefit from BF have less education, are mostly light and dark-skinned black people and are concentrated in the Northeast region of the country. These women also perform worse on NCD indicators such as having a higher incidence of being overweight and obese, having hypertension, using more tobacco, having a poorer perception of their health and having higher cholesterol levels when compared to non-beneficiary women.

These findings point to a positive and indirect evaluation of BF, since it seems that those who receive it also have worse health, as well as worse socioeconomic circumstances. Therefore, the importance of social programs in the form of income transfer is once again emphasized, as they are designed to increase the guarantee of social protection, addressing poverty and breaking its intergenerational cycle, and thus reducing social inequalities^17^.

Given this fact, it is internationally agreed that, to improve health and reduce mortality in the population, it is necessary to plan interventions that address social determinants of health^4^. Thus, PTCRs, by providing income to poor households, can reduce inequalities among beneficiary families^33,34^. Currently, BF is the largest PTCR not just in Brazil, but in the world, in relative and absolute terms^33,35,36^.

It is also emphasized that the cause of these disparities is multifactorial and is associated with low levels of education and income. Some studies have previously demonstrated these inequities for cardiovascular disease^14^, for example, and also for some of the most prevalent risk factors among women with low levels of education^11^. Additionally, they have shown the high prevalence of obesity^12^, usually associated with low-income populations and racial inequalities^13,37^.

Studying inequities becomes relevant because it reinforces the need to expand population subgroups’ access to health care, actions and programs. Women in Brazil generally receive lower wages and have unfavorable working situations, which reinforces historical gender inequality, and in turn, aggravates their health situation^15,16^.

This research points out worse health indicators among women that benefit from BF and shows the importance of taking ownership of the benefit granted to them, as they have been identified as more responsible and cautious^33,38^. Ultimately, this benefit can mitigate the disparities described here. This governmental action is based on the concept of positive discrimination, considered by Souza^18^ to be necessary in order to benefit more vulnerable populations. The goal is to reduce inequalities, such as those faced by poor and socially disadvantaged women, the profile of BF beneficiaries^19–21^.

Socioeconomic inequality is a factor that in itself leads to an increase in NCDs in low-income populations. Global analyses across countries suggest that living in a low-income country is associated with a marked risk of developing chronic diseases^4,5^.

Another point that reinforces the increase in NCDs in this particular population is that social determinants extrapolate biological mechanisms by generating living standards that reflect social inequities, ultimately causing problems that accumulate over one’s lifetime^4,22^. In addition, young women that are still in their reproductive age have significant risk factors and chronic health problems, and these conditions may determine poor reproductive outcomes and have repercussions for their children’s health through transgenerational transfer^39^. However, there are not many studies that focus on investigating these diseases in women of reproductive age^9,10^. They are even more scarce in the Brazilian context, showing the need to make this public health problem visible and to make advances toward a comprehensive approach to women’s health.

Finally, it is important to highlight monitoring and NCD surveillance that includes vulnerable populations. Specifically, the implementation of the PNS in 2013 that included the question about receipt of BF allowed for this type of analysis and information on risk and morbidity to be available for this group.

This study has some limitations, including losses in the collection of laboratory tests, making it necessary to use post-stratification weights to reduce representation bias. After these procedures, the PNS laboratory results can be estimated for the Brazilian adult population. Laboratory tests may also have been lost due to hemolysis and insufficient material. Thus, there were differences between the number of women who performed biological material collection and the number of women who answered the questionnaire. It is also worth noting that the risk factor indicators were self-reported and may have resulted in memory bias.

## Conclusion

The results of this study show that several NCD indicators perform worse among BF beneficiary women of reproductive age. It is worth noting that this is not a causal relationship, and points to the importance of BF as a marker of inequality among women. The BF program addresses the population with the greatest health needs, and therefore needs to be maintained in order to reduce health inequities.

It should be highlighted that the present investigation analyzed the prevalence of NCD indicators among women of reproductive age who benefit from BF for the first time in Brazil. This may support the view that BF is being applied appropriately, but mainly it demonstrates that groups in the worst social and economic situations have the worst health conditions. These data should be considered when defining the health priorities for the Brazilian population, especially with regard to women’s health.

## Figures and Tables

**Table 1:** Distribution of women between 18 and 49 years old that received and did not receive Bolsa Família, according to age, educational level, skin color and region of residence. Brazil, National Health Survey (PNS), 2014–2015.

		Bolsa Família						Total (n = 3,131)		
		Yes (n = 924)			No (n = 2,207)					
		N	%*	95%CI	N	%*	95%CI	N	%*	95%CI
Total		924	23.28	21.50 – 25.15	2.207	76.72	74.85 – 78.50			
Age (years)	18 to 29	252	31.78	27.80 – 36.04	835	43.57	40.73 – 46.45	1.087	40.83	38.44 – 43.26
	30 to 44	608	61.32	57.01 – 65.46	1.161	47.77	44.96 – 50.59	1.769	50.92	48.53 – 53.31
	45 to 49	64	6.91	5.10 – 9.29	211	8.66	7.33 – 10.21	275	8.25	7.13 – 9.54
Education (years)	0 to 8	464	49.57	45.31 – 53.82	434	16.17	14.38 – 18.13	898	23.94	22.12 – 25.87
	9 to 11	186	22.78	19.27 – 26.71	360	16.05	14.13 – 18.16	546	17.61	15.90 – 19.46
	12 or more	274	27.66	24.07 – 31.55	1.413	67.79	65.20 – 70.27	1.687	58.45	56.13 – 60.73
Skin color	White	191	26.65	22.73 – 30.98	842	51.08	48.26 – 53.90	1.033	45.4	42.98 – 47.84
	Dark-skinned black	97	10.75	8.43 – 13.61	167	8.19	6.70 – 9.99	264	8.79	7.49 – 10.29
	Light-skinned black	622	61.55	57.17 – 65.76	1.152	39.59	36.99 – 42.26	1.774	44.71	4.24 – 47.03
	Other	14	1.04	0.05 – 2.40	45	1.13	0.66 – 1.92	59	1.11	0.07 – 1.74
Region	North	294	11.62	10.15 – 13.27	635	7.6	6.92 – 8.33	929	8.53	7.90 – 9.21
	Northeast	469	50.19	45.93 – 54.45	612	21.45	19.71 – 23.31	1.081	28.14	26.41 – 29.94
	Southeast	73	26.77	22.18 – 31.92	371	45.6	42.66 – 48.57	444	41.21	38.65 – 43.82
	South	30	6.03	3.97 – 9.04	303	16.79	15.00 – 18.75	333	14.29	12.81 – 15.90
	Center West	58	5.39	4.09 – 7.06	286	8.56	7.53 – 9.72	344	7.82	6.97 – 8.77

95% CI: 95% confidence interval.

* Weighted frequency.

**Table 2:** Prevalence of risk factors for chronic non-communicable diseases (NCDs) in women between 18 and 49 years of age, according to whether or not they received Bolsa Família. National Health Survey (PNS) 2013, Brazil.

Risk Factors for NCDs	Received Bolsa Família				Total		p ^*^
	Yes (n = 924)		No (n = 2.207)		(n = 3.131)		
	%^**^	95%CI	%^**^	95%CI	%^**^	95%CI	
Body mass index (measured)							
Overweight	33.5	29.6 – 37.6	29.3	26.8 – 32.0	30.3	28.2 – 32.5	0.001
Obese	26.9	23.1 – 31.1	21.2	18.9 – 23.6	22.5	20.6 – 24.6	
Smoker							
Yes	11.3	9.0 – 14.1	8.2	6.8 – 9.8	8.9	7.7 – 10.3	0.029
Consumes fatty red meat							
Yes	30.3	26.2 – 34.9	25.5	23.0 – 28.2	26.6	24.5 – 28.9	0.056
Consumes sodas five or more days a week							
Yes	27.0	23.2 – 31.2	27.6	25.0 – 30.3	27.5	25.3 – 29.8	0.800
Consumes alcoholic beverages once a month or more							
Yes	13.4	10.6 – 16.9	18.2	16.1 – 20.6	17.1	15.3 – 19.1	0.022
Consumes beans five or more days a week							
Yes	75.0	71.5 – 78.1	65.5	62.8 – 68.0	67.7	65.5 – 69.8	< 0.001

95% CI: 95% confidence interval.

* Pearson′s χ^2^

** weighted frequency.

**Table 3:** Prevalence of access indicators and self-reported chronic non-communicable diseases (NCDs) in women between 18 and 49 years of age, according to whether or not they received Bolsa Família. National Health Survey (PNS) 2013, Brazil.

Risk factors for NCDs	Received Bolsa Família				Total		p*
	Yes (n = 924)		No (n = 2,207)		(n = 3,131)		
	%**	95%CI	%**	95%CI	%**	95%CI	
Self-evaluation of health							
Very good	61.6	57.6 – 65.5	75.3	72.9 – 77.5	72.1	70.1 – 74.1	< 0.001
Fair	32.2	28.6 – 36.01	22.3	20.1 – 24.6	24.6	22.7 – 26.6	
Poor	6.2	4.7 – 8.2	2.4	1.8 – 3.3	3.3	2.7 – 4.1	
Has medical or dental insurance							
Yes	5.6	3.8 – 8.1	37.1	34.3 – 39.9	29.8	27.5 – 32.1	< 0.001
Sought out health services in the past year							
Yes	21.0	17.7 – 24.8	20.3	18.0 – 22.7	20.4	18.5 – 22.5	0.720
Has been hospitalized in the past 12 months							
Yes	9.2	7.0 – 12.0	7.9	6.5 – 9.4	8.2	7.0 – 9.5	0.335
Self-reported hypertension							
Yes	13.4	10.7 – 16.5	10.3	8.7 – 12.0	11.0	9.6 – 12.5	< 0.001
Only during pregnancy	7.4	5.09 – 10.5	3.4	2.54 – 4.47	4.3	3.43 – 5.37	
Self-reported diabetes							
Yes	2.9	1.8 – 4.6	2.1	1.4 – 3.0	2.2	1.6 – 3.0	0.134
Only during pregnancy	2.5	1.2 – 5.0	1.2	0.6 – 2.1	1.5	0.9 – 2.3	
Self-reported high cholesterol							
Yes	8.3	6.32 – 10.9	9.8	8.2 – 11.7	9.5	8.1 – 11.0	0.328
Self-reported arthritis or rheumatism							
Yes	4.2	2.9 – 6.1	3.5	2.7 – 4.6	3.7	3.0 – 4.6	0.426
Self-reported renal insufficiency							
Yes	1.6	0.7 – 3.5	1.5	0.9 – 2.6	1.6	1.0 – 2.4	0.935

95% CI: 95% confidence interval.

* Pearson′s χ^2^

** weighted frequency.

**Table 4:** Laboratory results in women between 18 and 49 years of age, according to whether or not they receive Bolsa Família. National Health Survey (PNS) 2014 – 2015**, Brazil.

NCD laboratory indicators	Bolsa Família				Adjusted PR	95%CI
	Yes		No			
	%*	95%CI	%*	95%CI		
Renal insufficiency (ckdepi) GFR < 60	1.84	0.74 – 2.93	1.28	0.70 – 1.86	1.49	0.69 – 3.21
Total cholesterol (TC) ≥ 200 mg/dL	23.87	20.36 – 27.37	26.28	23.76 – 28.79	0.89	0.75 – 1.06
HDL cholesterol < 40 mg/dL	28.19	24.19 – 32.19	19.72	17.51 – 21.93	1.41	1.17 – 1.69
LDL cholesterol ≥ 130 mg/dL	13.24	10.47 – 16.00	13.89	11.89 – 15.89	0.92	0.72 – 1.19
Creatinine ≥ 1.3 mg/dL	1.10	0.18 – 2.02	0.75	0.28 – 1.22	1.61	0.55 – 4.71
Glycated hemoglobin ≥ 6.5 mg/dL	4.07	2.01 – 6.13	2.72	1.84 – 3.60	1.42	0.79 – 2.54
Anemia (hemoglobin < 12 g/dL)	14.73	11.79 – 17.66	12.13	10.28 – 13.99	1.20	0.93 – 1.55
Salt consumption (>75^th^ percentile)	25.26	20.94 – 29.58	24.13	21.28 – 26.99	1.03	0.85 – 1.26

NCD: noncommunicable disease; 95% CI: 95% confidence interval; PR: prevalence ratio adjusted for age; GFR: glomerular filtration rate; HDL: high density lipoprotein; LDL: low density lipoprotein

* weighted frequency.

** the laboratory data were processed after the PNS from 2013.
